# A Feasibility Analysis of an Application-Based Partial Initialization (API) Protocol for Underwater Wireless Acoustic Sensor Networks

**DOI:** 10.3390/s20195635

**Published:** 2020-10-02

**Authors:** Changho Yun, Suhan Choi

**Affiliations:** 1 Division of Ocean System Engineering, Korea Research Institute of Ships & Ocean Engineering (KRISO), 32, Yuseong-daero 1312beon-gil, Yuseong-gu, Daejeon 34103, Korea; sgn0178@kriso.re.kr; 2 Department of Mobile System Engineering, Dankook University, 152, Jukjeon-ro, Suji-gu, Yongin-si, Gyeonggi-do 16890, Korea

**Keywords:** initialization protocol, underwater wireless acoustic sensor network, partial initialization, application

## Abstract

Initialization methods for underwater wireless acoustic sensor networks (UWASNs) have been proposed as a subset of other network protocols under the simple assumption that all the nodes in the network can be initialized at once. However, it is generally time- and energy-intensive to initialize all nodes in a UWASN due to unstable underwater channel conditions. To improve network efficiency, we propose the Application-based Partial Initialization (API) protocol, which initializes only the same number of nodes as the number of activated nodes required to run a specific application. Reducing the number of active nodes is also particularly advantageous underwater since the replacement of batteries is costly. To the best of our knowledge, the API is the first approach that initializes nodes partially according to applications. Thus, we investigate the feasibility of the API for a UWASN by analyzing its performance via simulations. From the results, it is shown that the API provides similar data statistics compared with the conventional full initialization that initializes all nodes. Moreover, the API outperforms the full initialization in terms of the initialization time and message overhead performances.

## 1. Introduction

Underwater wireless acoustic sensor networks (UWASNs) are considered adequate communication infrastructures because acoustic communications can guarantee more reliable communication with a longer propagation distance than optical and radio-frequency (RF) counterparts [1] in underwater environments. Therefore, UWASNs have been adopted widely for diverse applications including scientific observation, the exploitation of ocean resources, disaster detection, military surveillance and subsea construction monitoring [2,3].

In our study, we focus on observation-based applications that deploy multiple nodes underwater and constantly obtain sensed data from them. By using the statistics of the data set sensed and collected by multiple underwater nodes, we can derive the desired information and predict future trends of underwater phenomena.

Let us consider two cases in terms of the method to obtain data from nodes. One is to obtain data from all the nodes in the network. We call this “Case 1”. The other is to obtain data from only some of them: “Case 2”. When the sensed data varies significantly depending on region and time, for example, when investigating ship accidents, searching for drowned persons, or engaging in military surveillance, all nodes must be activated as in Case 1. On the other hand, in the case of observing the marine environment (e.g., salinity or temperature) for long periods of time, the similarity of the sensed data can be large, so the number of active nodes can be reduced, as in Case 2.

Accordingly, if the statistics of the data set in Case 2 are similar to those in Case 1 within an allowable error range, it is not necessary to acquire data from all the nodes in the network. Obtaining data from only some nodes is equivalent to partially activating the nodes in the network. This implies that Case 2 can reduce the number of active nodes, as compared with Case 1. A smaller number of active nodes is especially advantageous in underwater environments where battery replacement is costly.

The number of active nodes can be an important factor in the sense that it determines the similarity of data statistics between the two cases. That is, the degree of similarity increases as the number of active nodes approaches the total number of nodes, and vice versa. If an application requires a high degree of data accuracy, the number of active nodes should be increased. On the other hand, if an application just predicts the overall trend with marginal data accuracy, the number of active nodes can be reduced. Therefore, the number of active nodes is application-specific and should be considered as a requirement or an important parameter for the UWASN.

Nodes can be activated and go to the ready state through an initialization process before an application starts. For this reason, we associate the application requirement (i.e., the number of active nodes) with initialization. More specifically, we initialize as many nodes as required to run an application, which is defined as “*partial initialization*”. The concept opposed to partial initialization is “full initialization”, which initializes all the nodes in the network.

In the literature, most initialization methods for a UWASN are a subset of a specific network protocol, as summarized in Table 1. Most of the initialization methods exist as an initial stage of a MAC protocol, as proposed in [4,5,6,7,8,9,10,11,12]. Moreover, they are a part of a routing protocol, as exemplified in [13,14,15], or part of a node discovery protocol [16]. Furthermore, they have been proposed under the simple assumption that all nodes in a network need to be initialized at once before the network transmits and receives data for applications.

To the best of our knowledge, there has been no study on a partial initialization approach. This motivates us to propose a new initialization protocol for a UWASN that partially initializes a UWASN according to the given application requirement.

In this paper, we propose the Application-based Partial Initialization (API), whose design principles are outlined as follows:

- Partial initialization. This protocol initializes as many nodes as required to execute a given application.
- Limited repetition. In a UWASN, communication failures frequently occur due to bad channel conditions, including natural or artificial noises as well as the Doppler effect [17]. This poor underwater environment makes it difficult for nodes to be initialized at once. Thus, the same process needs to be repeated in order to satisfy the application requirement.
- Simplicity. The method of initializing nodes is designed simply to be compatible with any network protocol. Accordingly, this protocol adopts the round-robin-based message exchange algorithm, which does not require scheduling, time synchronization, or retransmission.

Since there have been no API-like protocols for a UWASN, we analyze the feasibility of the API by the following two steps:

- First, we investigate whether the API can obtain data whose statistics are similar to data that requires full initialization (FI). To do this, we perform a simulation that generates a random data set of all nodes periodically and chooses only some nodes randomly according to the application requirement. At each period, we calculate the statistics of the accumulated data sets of the API and the FI, respectively, and check their similarity. Using the results, we can conclude that partial initialization can be used instead of full initialization, provided that the similarity in the data statistics between the API and FI converges and exists within the target range.
- Second, we check how much the API can improve in performance over the FI. Intuitively, we can predict that the API can guarantee better performance than the FI in some aspects since the API can have a smaller number of active nodes. However, it is difficult to determine the degree of which the API can outperform the FI since it varies depending on the performance metrics. We consider two performance metrics: (1) the initialization delay and (2) the message overhead. The initialization delay measures how long the initialization takes to satisfy the requirement of a given application. The message overhead calculates the amount of the messages required for initialization, which is closely related to the energy efficiency. Furthermore, we execute simulations extensively by varying the application requirement, the randomness of the data, and the bit error rate of the acoustic channels to check the performance superiority of the API to the FI.

From the feasibility study results, we show that the API not only provides similar data statistics compared to the FI, but can also improve overall network performance. Moreover, the API can be a more advanced initialization approach than conventional simple initialization methods for a UWASN in the sense that it partially initializes the network considering application requirements and promptly responds to poor underwater environments via repetitive initialization.

In addition, due to the partial initialization characteristic, the API can be favorable for any underwater applications requiring high energy efficiency. These applications periodically generate a volume of data from multiple under nodes for a long time like the Internet of Underwater Things (IoUT). These underwater applications are exemplified as follows [18]:

- The environmental observation-based applications that periodically measure various environmental information such as water quality, pollution, salinity, and temperature.
- The disaster prevention-based applications that monitor floods, earthquakes, and tsunamis in real time.
- The fisheries applications such as fish and shellfish farming.

We organize the rest of this paper as follows: In Section 2, we explain the network model applying the API. In Section 3, the algorithm of the API is described in detail. In Section 4, we analyze the performance of the API via extensive simulation tests in order to investigate its feasibility. Section 5 concludes this paper.

## 2. Network Model

This section describes the UWASN model for applying the API protocol. We consider a centralized topology that is efficient to gather data from multiple underwater nodes for observation-based applications. In addition, the centralized topology is capable of managing network resources easily and supporting reliable network operations compared to a decentralized topology (e.g., ad hoc).

The UWASN can be either two-dimensional or three-dimensional. In the two-dimensional UWASN, all nodes are located at the seabed or at an equal depth of water. Thus, horizontal communication is dominant. An example of the two-dimensional UWASN is a cluster-based sensor network composed of multiple sensor nodes centering on a cluster head [19,20]. The three-dimensional UWASN consisting of a buoy on the water surface and several underwater nodes located beneath the buoy is based on vertical communication as explained in [21].

**Figure 1:**
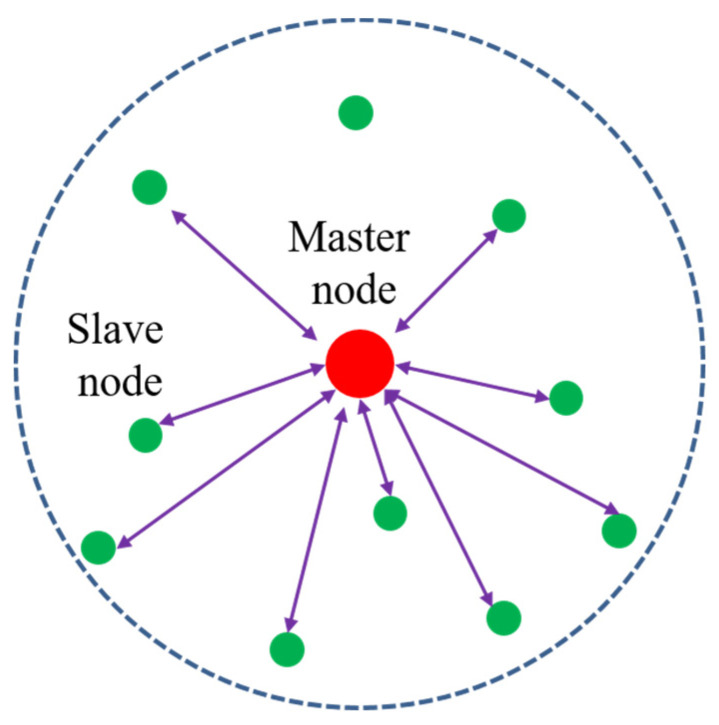
The architecture of the UWASN to apply the API protocol.

As shown in Figure 1, the UWASN consists of a master node and a number of slave nodes. The master node located at the center is responsible for controlling all the slave nodes, gathering data from them, and forwarding the data to a land base station. The master node also sends data from the land base station to the slave nodes. A slave node located within the master node’s coverage enters a network and exchanges data with other nodes under the control of the master node. A master node is exemplified as a cluster head, a buoy, or an underwater base station. Slave nodes can be either mobile (e.g., AUVs or underwater drones) or fixed (e.g., fixed sensors).

The UWASN is operated through two stages; one is the initialization stage, and the other is the operational stage. The API protocol is employed in the initialization stage. A master node manages the start and the end of each stage. When frequent network failures occur, and thus an ongoing application is no longer executable, the master node terminates the operational stage and returns to the initialization stage.

All the nodes of the UWASN are equipped with a digital acoustic communication module that provides the following common characteristics:

- The communication between a master node and slave nodes is based on a half-duplex scheme. That is, one acoustic channel in the frequency domain is used.
- A node can communicate with any nodes within its communication range by using an omni-directional antenna.
- Depending on the transmission power of acoustic signals, nodes can be connected with each other via either one-hop or multi-hop. In the initialization stage, it is assumed that any slave node can connect to a master node directly by sending signals with the maximum power.
- All nodes obtain sensing capability by having a sensor attached according to applications.

## 3. API Protocol

In this section, we explain the API protocol. The main idea of the API is that only the nodes required to run a specific application are initialized via the repetition of the same process by considering an underwater channel. In the API, we assume the following conditions:

- A master node already knows the information of slave nodes, including their identification number and the number of slave nodes.
- Network protocol (e.g., MAC, routing, etc.) information required for the operational stage is fully delivered to all slave nodes by the master node through the initialization message.
- Once the initialization stage is terminated, the operational stage follows based on the given network protocol, which is controlled by the master node.

The API is based on one-to-one initialization between a master and a slave node. To begin initialization, a master node transmits an initialization request message to a slave node. The slave node, which received the initialization request message without error, then completes initialization by sending an initialization response message to the master node.

Although a complex scheduling method can be applied in the initialization stage, a simple round-robin method where a master node sequentially initializes a slave node one by one is applied due to the following reasons:

- It clearly shows the performance difference between the API and the FI by excluding complicated collisional situations.
- It reduces the algorithm complexity and enables the API to be compatible by avoiding complex scheduling or time synchronization.
- It focuses on enhancing the initialization success rate by avoiding performance degradation due to both transmission and reception collisions.

**Figure 2:**
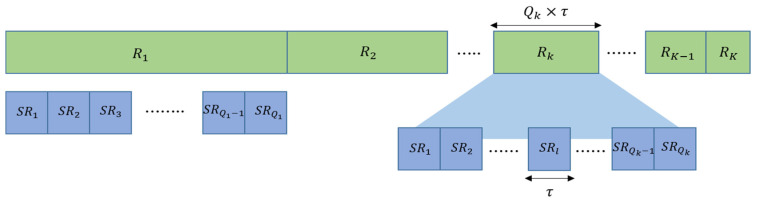
The illustration of the concept of a round and a sub-round in the API protocol.

Before explaining the algorithm, let us define several terminologies used in the API protocol as follows:

- Round. This is used to represent the repetition of the initialization process. As illustrated in Figure 2, a master node executes the one-to-one initialization with multiple slave nodes in one round. The index of a round is defined as k. k does not increase to infinity but can increase to the maximum value K (k≤K), where K is a network parameter and can be determined according to network conditions. Here, the $k_{th}$ round is simply denoted by $R_{k}$.
- Sub-Round. This represents the order of the one-to-one initialization. In a sub-round, a master node initializes a slave node. The index of a sub-round is defined by l.
- The number of sub-rounds of the $k_{th}$ round is defined as $Q_{k}$. As described earlier, a master node initializes as many slave nodes as the application requirement in the initialization stage. In $R_{1}$, the number of sub-rounds should be at least the application requirement in order to perform a sufficient number of one-to-one initializations. Thus, $Q_{1}$ is determined to be the same as the application requirement (i.e., the number of active nodes). If we define the number of slave nodes as N, the range of $Q_{1}$ is given as $1\leq Q_{1}\leq N$ and determined according to the application requirement. If the channel condition is good, $Q_{1}$ slave nodes can be initialized successfully in $R_{1}$. Otherwise, the application requirement cannot be satisfied in $R_{1}$ and the next round ($R_{2}$) must be performed. Let us denote the number of slave nodes that were successfully initialized in $R_{1}$ as ${\bar{Q}}_{1}$. The number of sub-rounds in $R_{2}$ can then be expressed as $Q_{1}-{\bar{Q}}_{1}$. If this is generalized to $R_{k}$, $Q_{k}$ can be expressed as:
  (1) $$Q_{k}=\{\begin{array}{cc} Q_{1}, & k=1\\ Q_{k-1}-{\bar{Q}}_{k-1}, & k>1 \end{array}$$
- All sub-rounds are set to have the same time duration as τ in order to avoid a complex scheduling considering spatial-time reuse. The value of τ is determined by considering the maximum propagation delay between the master and a slave node, the transmission delay, and the guard time. As shown in Figure 2, the time duration of a round differs from each other because it depends on the number of sub-rounds. Hence, the time duration of $R_{k}$ can be obtained from the equation: $R_{k}=\;Q_{k}\times \tau$.
- All parameters defined for the API are summarized in Table 2.

In the API, a master node controls overall network initialization. A master node performs the following procedures in one round:

(1) At the start of a round, a master node randomly chooses $Q_{k}$ slave nodes among uninitialized slave nodes and activates the selected slave nodes via a one-by-one initialization process. The number of selected slave nodes and the selected slave nodes vary according to the initialization round. In the case of $R_{1}$, a master node determines the number of sub-rounds $Q_{1}$ as the same as the application requirement.
(2) The master node then starts initializing slave nodes sequentially during $Q_{k}$ sub-rounds. At each sub-round, the master node sends an initialization request message to one of the selected slave nodes and waits for the initialization response message from the slave node during τ. If the master node receives an initialization response from the slave node before the expiration of that sub-round, it adds the slave node to the initialization success list. Otherwise, the master node confirms that the slave node failed to be initialized in that round. This one-to-one initialization is performed in ascending order of the slave node index.
(3) At the end of the round, the master node counts the number of slave nodes that were successfully initialized during $R_{k}$, which is denoted by ${\bar{Q}}_{k}$. Additionally, the master node calculates the accumulated number of slave nodes that have been successfully initialized until $R_{k}$, denoted by $N_{k}$. $N_{k}\;$is expressed as $N_{k}=\sum_{i=1}^{k}{\bar{Q}}_{i}$. The master node then checks whether the number of accumulated slave nodes that are successfully initialized satisfies the application requirement (i.e., $N_{k}\geq Q_{1}$).
(4) If the application requirement is satisfied, the master node terminates the initialization stage and goes to the operational stage.
(5) Otherwise, the master node increases the round index by 1 and checks whether the index exceeds the maximum number of rounds, K.
(6) If the increased index value exceeds K, the master node also terminates the initialization stage. This extreme case implies that the underwater channel is disabled and a UWASN failure occurs. In this case, the network initialization process needs to be restarted when the channel condition is good.
(7) Otherwise, the master proceeds to the next round and goes to Step 2.

The pseudo code for the API algorithm for a master node is specified in Algorithm 1.
**Algorithm 1**. The pseudo code of the algorithm for a master node.1:**for**k=1:1:K2: **if**
k=1 then3:  Set $Q_{1}$ according to the application requirement.4: **else**5:  Set $Q_{k}=Q_{k-1}-{\bar{Q}}_{k-1}$
6: **end if**7: Randomly select $Q_{k}$ slave nodes among uninitialized slave nodes.8: Set ${\bar{Q}}_{k}=0$.9: **for**
$j=1:1:Q_{k}$
10:  Send an initialization request message to an initialized slave node.11:  Wait for an initialization response message from the slave node during τ.12:  **if** the initialization response message is successfully received then13:   Set ${\bar{Q}}_{k}={\bar{Q}}_{k}+1$.14:  **end if**15: **end for**16: Calculate $N_{k}$.17: **if**
$N_{k}\geq Q_{1}$ then 18:Terminate the initialization stage, and break the for-loop19:**else**20:Go to Step 2. 21:**end if**22:**end for**

The procedure of a slave node is reactive and simple. That is, if a slave node receives an initialization request message successfully (i.e., decodable without errors) and confirms that it is the destination node specified in the initialization request message, then it immediately generates and transmits an initialization response message to the master node.

## 4. Feasibility Test of the API Protocol via Simulations

### 4.1. Feasibility Test of Partial Initialization

In this section, we investigate the feasibility of the partial initialization of the API before verifying the performance of the API via simulations. This is because the API can be of no use if the data statistics obtained by applying the API are not similar enough to those obtained by applying the FI.

#### 4.1.1. Simulation Operation and Conditions for Testing the Feasibility of the API Protocol

The simulation is built using MATLAB software and its operation is as follows:

- The simulation considers the uplink data transmission between a master node and several slave nodes in the operational stage.
- All the slave nodes generate random data of which value is randomly distributed according to the given data distribution.
- In the beginning of the simulation, some of them are randomly chosen according to the given application requirement. The application requirement is the ratio of the number of successfully initialized slave nodes to the total number of slave nodes in the network, which is also referred to as the initialization rate.
- The selected slave nodes are then put in the operational stage, and thus transmits data to a master node with respect to simulation time. It is assumed that there is no packet loss due to channel contentions.
- Depending on the given BER, an error may occur in the data transmitted by the slave node.
- This same data transmission process is repeated up to the given number of transmissions at the end of a simulation.
- At each transmission, the statistics of the accumulated data until current transmission for both the API and the FI are driven by the master node, respectively. By using the obtained statistics of both cases, the similarity is also obtained. The pattern of similarity is then checked.
- Simulations are executed case by case by changing the number of slave nodes, the BER, the type of data randomness, the initialization rate, and the standard deviation of data.

**Figure 3:**
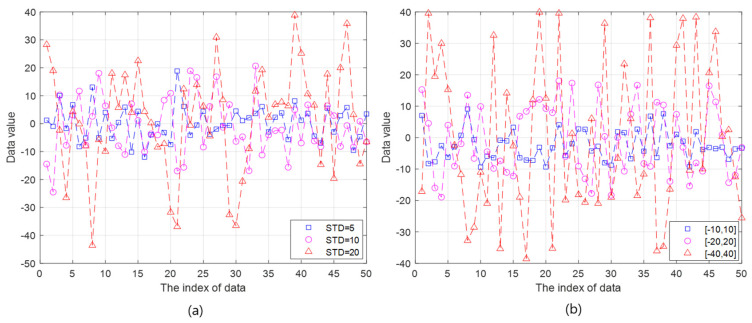
The illustration of generated data to check the feasibility of partial initialization. (**a**) The random data with normal distribution. (**b**) The random data with uniform distribution.

The similarity is determined by using the mean square difference (MSD) between the standard deviations (STDs) of accumulated data obtained by the API and that by the FI. We denote the STDs of the API and the FI by $STD_{API}$ and $STD_{FI}$, respectively. We then calculate the MSD by ${\left(STD_{API}-STD_{FI}\right)}^{2}$. The similarity, denoted by S, can be expressed as $S=\frac{1}{MSD+1}\;\left(0\leq \;S\leq 1\right)$ [22]. The similarity increases as the value of S approaches 1, and vice versa. The simulation conditions are given as follows:

- The value of BER is $5\times 10^{-4},10^{-3},\;\mathrm{and}\;10^{-2}$.
- The number of transmissions is 1000.
- The number of slave nodes is 10, 30, and 50.
- The initialization rates are set to 10%, 30%, 50%, 70%, and 100%. The FI is nothing, but the case of the initialization rate of 100%, which implies that all the slave nodes need to be initialized.
- It is difficult to predict the data model because the characteristics of the generated data vary depending on the underwater applications. In addition, the randomness of data distribution may further increase due to the unreliability of underwater acoustic communications. Therefore, the normal distribution is primarily employed in our simulations in consideration of the following reason. Based on the Central Limit Theorem (CLT), the measured data (or random variable) in underwater wireless networks is expected to follow the normal distribution with high probability because the measured random variable can be the sum of many independent random variables which were affected by many different natural phenomena in underwater. As shown in Figure 3a, the generated data for the simulations follows a normal distribution with the mean value of 1 and the STDs of 5, 10, and 20.
- In addition, the uniform distribution is also applied in simulations in order to investigate the feasibility of the API according to the type of data distributions. The uniform distribution is a typical probability density function(pdf) which is different from the normal distribution, is also simple and easily analyzed after obtaining the simulation results. Another data following a uniform distribution are in the range of [−10,10] (i.e., STD≈5.77), [−20,20] (i.e., STD≈11.54), and [−40,40] (i.e., STD≈23.09), as illustrated in Figure 3b.

#### 4.1.2. Results

**Figure 4:**
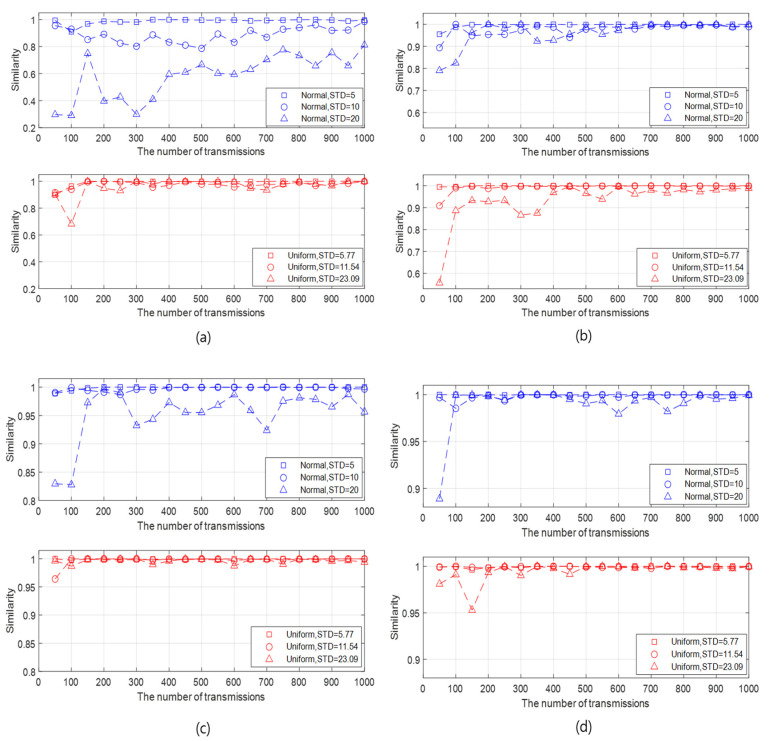
Similarities according to the number of transmissions for different data type, initialization rate, and STDs when the BER is $10^{-3}$ and the number of slave nodes is 30. (**a**) Initialization rate = 10%, (**b**) Initialization rate=30%, (**c**) Initialization rate = 50%, and (**d**) Initialization rate = 70%.

**Figure 5:**
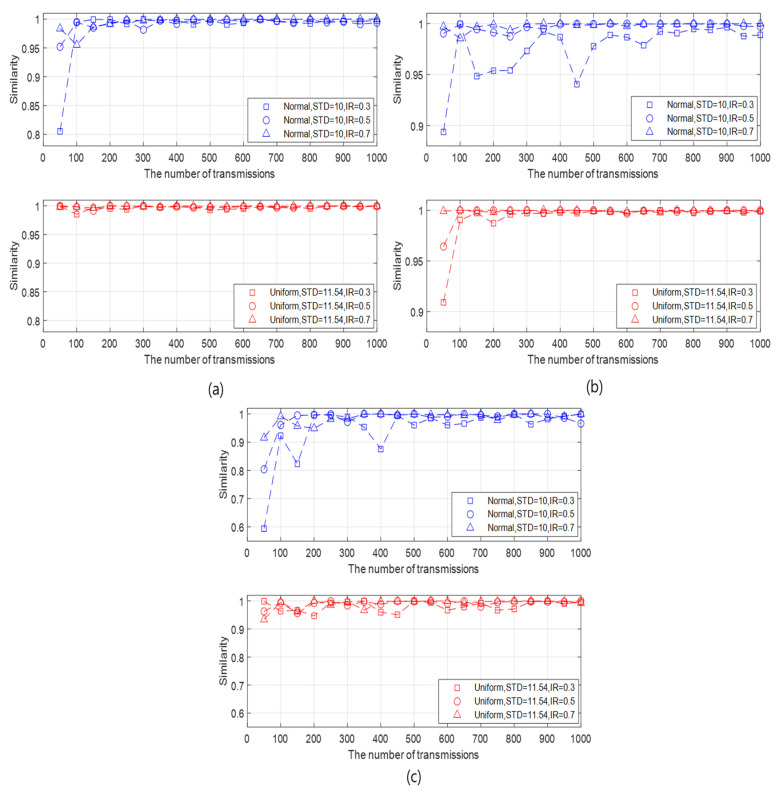
Similarities according to the number of transmissions for different data type, initialization rate, and the BER when the STD of the generated data with normal distribution and that with uniform distribution are respectively 10 and 11.54, and the number of slave nodes is 30. (**a**) BER = 5 $\times \;10^{-3}$, (**b**) BER = $10^{-3}$, and (**c**) BER = $10^{-2}$.

**Figure 6:**
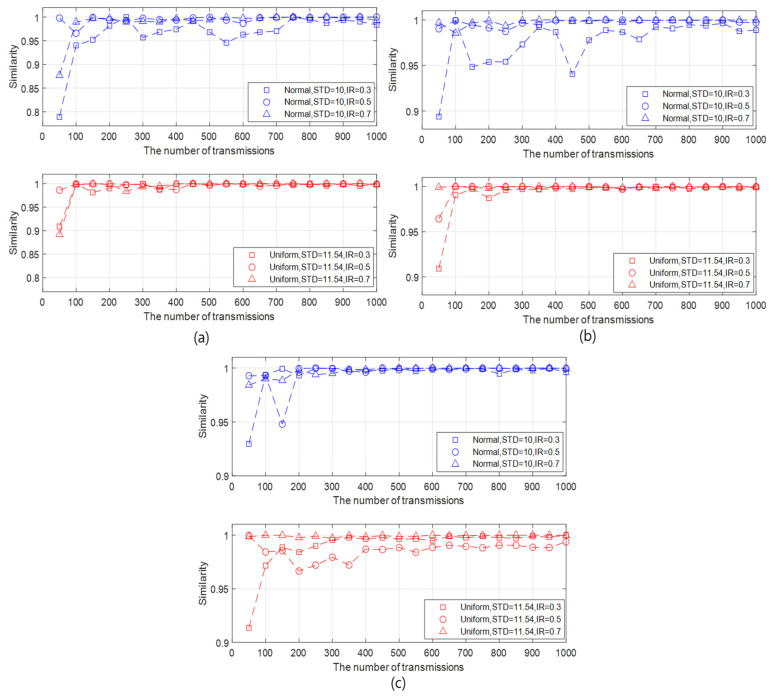
Similarities according to the number of transmissions for different data types, initialization rates, and amounts of slave nodes when the STD of the generated data with normal distribution and that with uniform distribution are, respectively, 10 and 11.54, and the BER is $10^{-3}$. (**a**) The number of slave nodes = 10. (**b**) The number of slave nodes = 30. (**c**) The number of slave nodes = 50.

We check the similarity between the FI (at the initialization rate of 100%) and the API (at initialization rates (IRs) of 10%, 30%, 50%, and 70%). We analyze the similarity results in the aspect of the initialization rate, the STD of the generated data, the BER, and the number of slave nodes. First, we analyze the similarity in the view of the initialization rate and the STD of the generated data as follows:

- It can be intuitively predicted that the higher similarity is guaranteed as the initialization rate becomes higher and the STD of the generated data set is lower. This prediction is confirmed in various cases by changing the STD of the generated data and the initialization rate, as illustrated in Figure 4a–d.
- As the number of transmissions increases, the overall similarity pattern fluctuates in the beginning and then converges after a certain number of transmissions. The larger the initialization rate, the smaller the number of transmissions where the similarity converges.
- The similarity increases as the initialization rate increases under the given data type and STDs. In addition, as the initialization rate increases, the variation of the similarity with respect to the number of transmissions decreases, as well as the effect of the STD on the similarity is weak. This result is due to the number of samples for which data statistics are obtained. When the initialization rate is high, the number active nodes also increases, which implies the increase of the number of samples. As more samples are accumulated, the similarity between the data statistics of the API and the FI also increases.
- Compared with the data with normal distribution, the data with uniform distribution guarantees slightly high similarity in most cases. This result is confirmed by Figure 4, Figure 5 and Figure 6. However, the similarity difference between two data types is unremarkable except the case where the initialization rate is very low and the standard deviation of the generated data is high, as shown in Figure 4a. When the initialization rate is low, the number of samples also decreases. In this case, the probability, that the data obtained from the active nodes by the API are near the mean, can be high in the normal distribution. This can increase the difference between the data obtained by the API and that by the FI since the FI obtains the data from all nodes. On the other hand, in the uniform distribution, the data are evenly spread depending on the standard deviation. Thus, the difference between the data by the API and that by the FI in the uniform distribution is less significant than that in the normal distribution. This leads to a similarity difference between the two distributions in the case.
- It can be seen that, except for the case where the initialization rate is very low, say 10%, the similarity is at least 90% under the given data type and STDs. Although it is confirmed that the API can be feasible to the given data type based on the simulation results, it cannot be generalized that the API can be effectively applied to all data models. Depending on the underwater application, there can be other data distributions for which the API do not operate properly, and thus the network must be initialized close to the FI (full initialization) by increasing the number of active nodes.

Second, the similarity results are analyzed in the aspect of the BER as follows:

- It can be intuitively predicted that the higher similarity is guaranteed as the BER is lower. This prediction is checked by varying the BER and the initialization rate, as illustrated in Figure 5a–c.
- As the BER decreases, the similarity becomes higher and more fluctuated according to the number of transmissions.
- The similarity of the generated data with normal distribution is lower than that with uniform distribution as the BER increases.
- However, the similarity is over 90% after a certain number of transmissions even when the BER is $10^{-2}.$
- As a result, even if a transmission failure occurs due to a slightly poor channel condition, it can be seen that the data statistics of the API and the FI have similarity.

Third, the similarity results are investigated in the view of the number of slave nodes as follows:

- As the number of slave nodes increases, the amount of data to be applied to the data statistics also increases. It is shown that the increase of the number of slave nodes affects the similarity differently according to the type of data.
- In the generated data with normal distribution, the higher the number of slave nodes, the faster the similarity reaches 90%. In addition, as the number of slave nodes increases, the similarity also increases, as shown in Figure 6a–c.
- On the contrary, in the generated data with uniform distribution, the similarity variation with respect to the number of slave nodes is unremarkable.
- As a result, the number of slave nodes can affect the similarity differently depending on the type of data distribution. In addition, if the number of slave nodes has influence on the similarity, the similarity increases as its value increases.

### 4.2. Performance Analysis of the API Protocol

In this section, we analyze the performance of the API and compare it with that of the FI via simulations. Two performance metrics are considered: (1) initialization delay and (2) message overhead. The initialization delay shows us how much the API can reduce the time needed to initialize the network compared with the FI. The message overhead informs us of how much the API can improve energy efficiency over the FI because the energy spent in transmission mode is much higher than that in reception mode or idle mode. Thus, the more messages that are transmitted, the more energy is spent.

#### 4.2.1. Simulation Operation and Conditions for Analyzing the Performance of the API Protocol

The simulation is built using MATLAB software and its operation is explained as follows:

- The simulation considers the initialization stage between a master node and a number of slave nodes.
- The position of a master node and slave nodes are randomly determined in the beginning of a simulation. It is assumed that the master node and any slave node can be connected directly in the initialization stage.
- A master node and slave nodes operate based on the initialization process as specified in Section 3.
- In the simulation, an initialization process between a master node and slave nodes can be executed up to K times. K is given as the same as the number of slave nodes. This is determined based on the assumption that at least one slave node can be initialized in one round such that as many rounds as the number of all the slave nodes can be iterated in the worst case.
- At the end of one process, the master node calculates the initialization success rate, and compares it to the given application requirement.
- Simulations are executed case by case with respect to the initialization rate and bit error rate (BER).
- For one case with a fixed BER and initialization rate, 10,000 simulation tests are performed, and the average values of the performance metrics are derived. After performing the simulation tests in the same way by changing the BER and the initialization rate, the performance of the API, and the FI are analyzed and compared with each other.

The parameters used in the simulations are as follows:

- The number of simulation experiments per case is 10,000.
- The number of slave nodes is 100.
- The maximum number of initialization repetitions is 100.
- The initialization rate is 0.2:0.2:1.0.
- The length of initialization messages is 100 bits.
- The BERs are $7\times 10^{-3},\;5\times 10^{-3}$, 3 $\times 10^{-3},\;10^{-3},$ and 0.

#### 4.2.2. Initialization Time

First, we investigate the initialization time of the API and the FI. In Section 3, the time duration of a sub-round is fixed as τ, and thus that of a round is represented as a multiple of sub-rounds. In $R_{1}$ (i.e., the first round), the maximum number of sub-rounds for initialization is $Q_{1}$ (i.e., the number of sub-rounds). $Q_{1}$ is determined by multiplying the number of slave nodes by the initialization rate (i.e., the application requirement).

If the application requirement is not satisfied in $R_{1}$, $Q_{2}$ sub-rounds will be conducted for initialization in $R_{2}$. If this is generalized to the case that the initialization is completed in $R_{k}$, the initialization time can be expressed as $\sum_{i=1}^{k}Q_{i}.$ Thus, the initialization time can be also expressed as a multiple of sub-rounds. This implies that a sub-round can be used as a unit to represent the initialization time metric.

**Figure 7:**
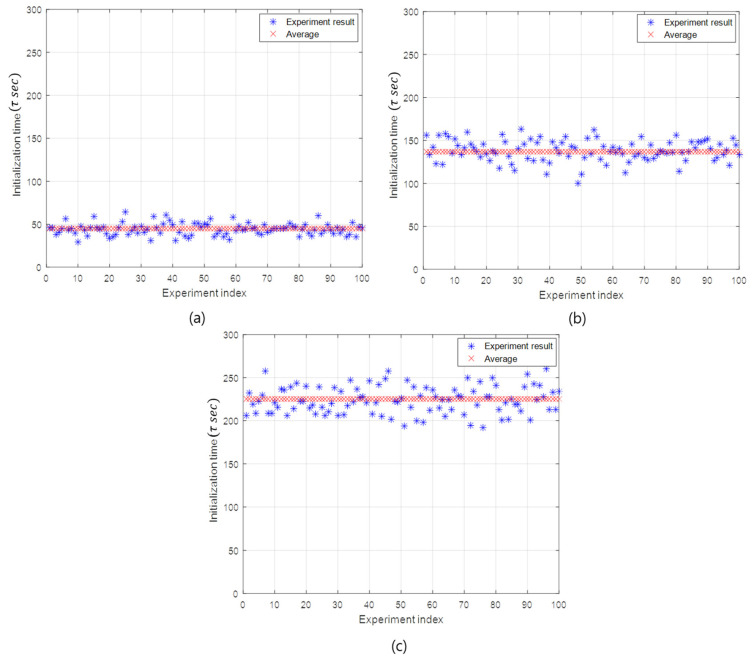
The initialization time of the API and the FI. (**a**) The API with the initialization rate 20%. (**b**) The API with the initialization rate 60%. (**c**) The FI with the initialization rate 100%.

Figure 7 illustrates the initialization time according to the simulation experiment index in the case of the BER of $3\times 10^{-3}$ and the initialization rates of 20%, 60%, and 100%. The initialization time is distributed around the average value, as shown in Figure 7. As the initialization rate increases, the initialization time also increases in order to initialize more slave nodes.

**Figure 8:**
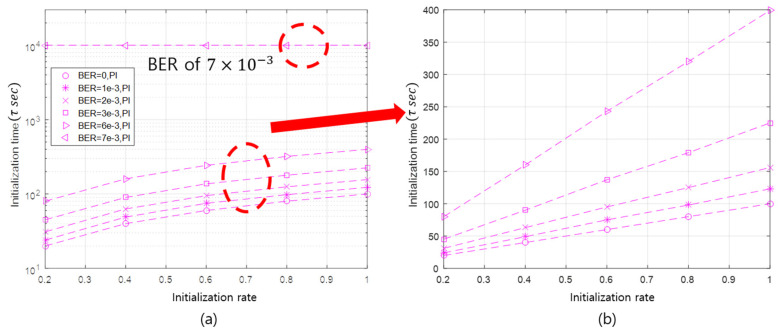
The initialization time of the API according to BER and the initialization rate.

In addition, the initialization time is linearly proportional to the initialization rate. When the initialization rate is 20%, the average initialization time takes about 45 sub-rounds. In the case of the initialization rates of 40%, 60% 80%, and 100%, the corresponding average initialization times are 90 (=2×45), 137 (≈3×45), 179 (≈4×45), and 225 (=5×45) sub-rounds, respectively. The linearity of the initialization time with respect to the initialization rate can be remarkably seen in Figure 8b. This linearity is originated from the property that the API selects as many slave nodes as the initialization rate.

This linearity persists until the BER deteriorates to a point where the communication between the master and slave nodes is very unreliable. Under the simulation conditions, when the BER value is $7\times 10^{-3}$, the linearity is broken, as illustrated in Figure 8a. This result can be predicted via simple calculation. The packet error rate (PER) can be expressed as $\mathrm{PER}=1-{\left(1-BER\right)}^{L}$, where L is the number of bits of a packet. By applying the PER equation, when the BER is $7\times 10^{-3}$ and L is 100 bits, the PER is approximately 0.5. From the simulation results, when the PER exceeds 50%, communication failure between a master node and a slave node is obvious. In this case, the initialization satisfying the application requirement cannot be realized, even if the initialization process is performed up to the maximum number of initialization repetitions. If the length of the packet is adjusted, the value of BER, which causes communication failure, will be also changed.

This linearity can be considered as a characteristic of the API that adaptively initializes nodes according to the initialization rate. Using this linearity, it is possible to roughly estimate the initialization time of the API according to the initialization rate in a specific channel state (e.g., BER).

**Figure 9:**
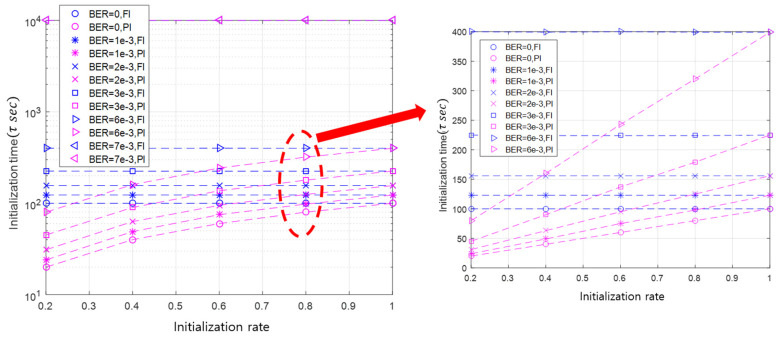
The initialization time of the API and the FI according to BER and the initialization rate.

Figure 9 shows the comparison of the initialization time between the API and the FI. Because the FI needs to initialize all slave nodes, its initialization time performance is unrelated to the initialization rate. Therefore, as the initialization rate increases, the initialization time of the API approaches that of the FI, which is shown in Figure 9.

The initialization time of the API and the FI deteriorates as BER increases. This is because a slave node does not receive the initialization request message from the master node, and the master node does not successfully receive the initialization response message sent by the slave nodes. Moreover, as the BER increases, the initialization is delayed due to frequent communication failures, and the performance gap between the API and the FI increases.

#### 4.2.3. Message Overhead

The one-to-one initialization between a master and a slave node takes up to two messages during one sub-round. The number of messages of a sub-round is 1 when the master node sends an initialization request message, but the slave node cannot receive the message. On the other hand, the number of messages is 2 when the slave node successfully receives the initialization request message, and it sends back an initialization response message to the master node. If the channel condition is good, two messages are transmitted in a sub-round. Otherwise, only one message is sent.

As there are $Q_{k}$ sub-rounds in $R_{k}$, the maximum number of messages that can occur in the round $R_{k}$ is $2\times Q_{k}$, and the minimum number of messages in $R_{k}\;$is $Q_{k}$. We define the message overhead as the number of all messages sent until the initialization is finished. Thus, the message overhead can be obtained by adding up all the generated messages up to the round of successful initialization. If this is generalized to the case where the initialization is completed in the $k_{th}$ round $R_{k}$, the message overhead can be calculated by $\sum_{j=1}^{k}\sum_{i=1}^{Q_{i}}M_{ji}$, where $M_{ji}$ is the number of messages sent in the $i_{th}$ sub-round in the $j_{th}$ round such that $M_{ji}\;$is 1 or 2.

**Figure 10:**
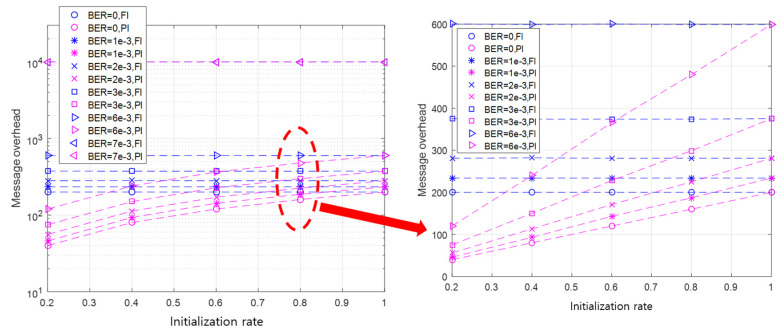
The message overhead of the API and FI according to BER and the initialization rate.

Using the message overhead calculation formula, the message overhead can also be expressed as the number of sub-rounds. This implies that the linearity of the initialization time according to the initialization rate can also be applied to the message overhead performance. The linearity is verified, as shown in Figure 10, where the API shows the linearity of performance according to the initialization rate, provided that the BER is not high enough to cause communication failures. From the results in Figure 10, it can be concluded that the performance pattern of the initialization time and message overhead according to the initialization rate and BER is similar.

After comparing the message overhead of the API to that of the FI, it is confirmed that, as the initialization rate increases, the message overhead of the API approaches that of the FI. In both the API and the FI, the message overhead performance deteriorates as the BER increases, and the performance gap between the API and the FI also becomes larger. This implies that the larger the BER and the higher the initialization rate, the more messages occur during initialization.

In conclusion, it is verified through our simulations that the API outperforms the FI with respect to both initialization time and message overhead. Furthermore, the performance difference between the API and the FI becomes remarkable when an application is run with a low initialization rate in a poor channel condition.

## 5. Discussion

Underwater is an unattended area such that battery replacement is costly. If an underwater network can be managed with only a subset of active nodes, we can extend the average battery life of the nodes. The number of required active nodes for an application varies depending on the type of applications. Thus, executing a specific application can be considered a requirement. Instead of initializing all the nodes in the underwater network, it would be more efficient to initialize as many nodes as required for an application. For this purpose, we proposed the application-based partial initialization (API) protocol in this paper. To the best of our knowledge, the API is the first initialization-only protocol that initializes only a portion of the nodes according to applications, and repeats the same initialization process until the application requirement is satisfied by considering unreliable underwater acoustic channel conditions. To apply the API for a UWASN, we also investigated its feasibility via simulations. We checked the similarity of data statistics between the API and the conventional FI (full initialization). The results showed that the similarity fluctuates in the beginning but saturates to 90% as the data accumulates. In addition, we analyzed the performance of the API and compared it to that of the FI. As a result, the initialization time and message overhead performances are noticeably improved when the FI is replaced by the API. The worse the channel is, and the smaller the application requirement (i.e., the required number of active nodes) is, the more remarkable the guaranteed performance superiority of the API is compared to the FI. Therefore, considering the bad channel conditions and high energy-efficient requirements of underwater networks, the API can be effectively applied to a UWASN since it is compatibly applied to any protocol. Moreover, the concept of the API can be employed to the IoUT where massive data sensing and gathering is necessary under energy-restricted underwater environments, and various applications require different levels of partial initialization.

## Figures and Tables

**Table 1:** Summary of previous works on underwater initialization methods.

Previous Work	Main Protocol	Initialization Method	
		Channel Access	Procedure
Park et al. (2007) [5]	MAC	Random access	SYNC broadcast among nodes
Ng et al. (2010) [6]		Undefined	Corresponding message broadcast among nodes
Yang et al. (2011) [7]		Undefined	Short INIT flooding from sink to sensors under tree topology
Khalil et al. (2012) [8]		Random access + back-off	An initiator broadcasts Pilot, and others send resend or success reply.
Kleunen et al. (2012) [9]		Scheduled	Corresponding message broadcast among nodes
Yun et al. (2013) [4]		TDMA	Corresponding message broadcast among nodes
Santos et al. (2016) [10]		TDMA	An initiator broadcasts SYNC, and others send ASYNC.
Morozs et al. (2017) [11]		Scheduled	Ping broadcast among nodes
Zhuo et al. (2018) [12]		Scheduled	An initiator broadcasts IRES, and others send IRP.
Sozer et al. (2000) [13]	Routing	Undefined	An initiator broadcasts Polling, and others send Response.
Basagni et al. (2015) [14]		Undefined	Hello flooding
Kim et al. (2017) [15]		Undefined	Hello flooding
Patil et al. (2011) [16]	Node discovery	ALOHA	Corresponding message broadcast among nodes

**Table 2:** The description of parameters for the API protocol.

Parameters	Description
N	The number of slave nodes
K	The maximum number of repetitions (or rounds)
k	An index of a round (1≤k≤K)
$R_{k}$	The $k_{th}$ round
$Q_{k}$	The number of sub-rounds in $R_{k}$
${\bar{Q}}_{k}$	The number of slave nodes that are successfully initialized in $R_{k}$
l	An index of a sub-round
$SR_{l}$	The $l_{th}$ sub-round
τ	The time duration of a sub-round
$N_{k}$	The accumulated number of slave nodes that are successfully initialized until $R_{k}$
